# Smoking, haptoglobin and fertility in humans

**DOI:** 10.1186/1617-9625-1-2

**Published:** 2002-01-15

**Authors:** N Bottini, A Magrini, J MacMurray, E Cosmi, M Nicotra, F Gloria-Bottini, A Bergamaschi

**Affiliations:** 1 The Burnham Institute, 10901 North Torrey Pines Road, La Jolla, CA 92037, USA; 2 Division of Occupational Health Medicine, Department of Biopathology and Imaging Diagnostics, University of Rome, Tor Vergata, School of Medicine, Rome, Italy; 3 Department of Medical Genetics, City of Hope National Medical Center, Duarte, California; 4 Institute of Obstetrics and Gynecology, University of Rome La Sapienza, School of Medicine, Rome, Italy; 5 Division of Medical Statistics, Department of Biopathology and Imaging Diagnostics, University of Rome Tor Vergata, School of Medicine, Rome, Italy

## Abstract

A prospective study on two samples of consecutive puerperae (total n° 667) from two populations has been carried out in order to investigate the possible effect of smoking habit on relationship between fertility and haptoglobin phenotype.

In both populations the negative association previously reported between age of pueperae and Haptoglobin *1/*1 phenotype is present only in women with smoking habit pointing to an interaction between Hp and smoke on human fertility. This suggests that the effects of smoke on fertility are dependent on the Hp phenotype.

## Introduction

We have recently reported that the distribution of haptoglobin phenotypes in two samples of consecutive healthy puerperae depends on the age of women, suggesting that women with Hp*1/*1 phenotype reproduce at an earlier age and have higher natural fertility potential than women with other Hp phenotypes [1]. Since Hp down-regulates the inflammatory response, and since it has been shown that Hp messenger RNA is expressed at implantation-stage endometrium [2], it is likely that Hp plays an important role in the mediation of maternal reaction against the blastocyst and that this effect depends on Hp phenotype. Recently it has been shown that tobacco smoke increases the serum level of Hp [3]. This prompted us to reconsider our data on healthy puerperae to search for possible interaction between smoking habit and Hp phenotype on female fertility.

## Subjects and Methods

We reviewed the clinical records of two series of healthy puerperae previously described [1], from the cities of Penne (mean age 27 years, SD 5) and Rome (mean age 28 years, SD 6). Reliable information on smoking habit during pregnancy was obtained for 313 women in Penne and for 354 women in Rome. We have also reviewed a series of newborn infants in whom the appearance of Hp electrophoretic pattern at three days of life had been studied [4]. Haptoglobin phenotype was determined by starch gel electrophoresis as previously described [1]. Three way contingency tables were analyzed by a log linear model according to Sokal and Rohlf [5]. Probabilities were combined according to Sokal and Rohlf [5]. Chi-square test was performed using the Statistical Package for the Social Science (SPSS).

## Results

Table 1 shows the distribution of Hp phenotypes in relation to maternal age and smoking habit. The association of maternal age with haptoglobin phenotype previously described [1] is present only among women with a smoking habit. The phenomenon is concordant in the two populations considered. The deviation from Hardy-Weinberg expectation of Hp phenotypes distribution previously described in women aged less than 24 [1] is present only among women with a smoking habit.

**Table 1:** Per cent distribution of Hp phenotypes in consecutive healthy puerperae

		Rome				Penne			
		Hp phenotypes				Hp phenotypes			
		*1/*1	*2/*1	*2/*2	n°	*1/*1	*2/*1	*2/*2	n°
Smoking	< 24 yrs(A)	25.0	40.0	35.0	40	42.8	42.8	14.3	7
	≥ 24 yrs(B)	13.8	42.6	43.6	94	8.3	41.7	50.0	24
Not smoking	< 24 yrs(C)	15.8	44.7	39.5	38	18.6	48.6	32.8	70
	≥ 24 yrs(D)	14.2	43.4	44.0	182	10.4	45.3	44.3	212
									
**Three way contingency table analysis by a loglinear model**									

x = Hp phenotype									
y = age									
z = locality									
	*1/*1 vs other Hp phenotypes			*1/*1 vs *2/*2 phenotype					

	G	df	p	G	df	p			
SMOKING									
xyz interaction	1.453	1	NS	2.241	1	NS			
xy association	6.399	2	< 0.05	7.686	2	< 0.02			
NON-SMOKING									
xyz interaction	0.454	1	NS	0.662	1	NS			
xy association	3.275	2	NS	4.623	2	NS			
									
Comparison between Hp*1/*1 with smoking habit vs other women									

age < 24 yrs vs age ≥ 24 yrs (Rome plus Penne)									
Chi square test of independence		df	p						
8.82		1	< 0.005						
									
**Deviation from Hardy-Weinberg expectation**									

		Rome				Penne			
(A)		p < 0.02				p < 0.02 (combining probabilities p < 0.005)			
(B)		p NS				p NS			
(C)		p NS				p NS			
(D)		p NS				p NS			
**Hp gene frequencies**		Hp*1				Hp*2			
Rome		0.362				0.638			
Penne		0.356				0.644			

Table 2 shows the proportion of Hp*1/*1 phenotype in infants with detectable Hp pattern in the third day of life. The proportion of Hp*1/*1 is higher in infants from smoking mothers than in infants from mothers who did not smoke. The difference, however, does not reach the level of statistical significance.

**Table 2:** Proportion of Hp*1/*1 phenotypes among infants with detectable Hp electrophoretic pattern in the third day of life. The relationship with maternal smoking habit

	Smoking mothers	Non-smoking mothers
Proportion of Hp*1/*1 phenotype	21.4%	12.7%
n° of infants with a detectable pattern of serum Hp	56	173
Significance of difference	p ~ 0.10	

## Discussion

As discussed by Gimelfarb and Bottini [6], the number of children produced by a modern woman is much lower than her reproductive capacity and, hence, does not reflect her natural fertility. On the other hand, if there is a limit to the number of children that the women will have, women who have higher fertility will reach this limit at an earlier age than women who have lower fertility. Consequently, genotypes associated with high fertility should be represented in higher proportion among younger pregnant women than among older ones. Our data show that Hp*1/*1 women have a higher natural fertility only in association with smoking habit; thus suggesting that the effect of smoke on fertility is dependent on the Hp genotype.

Haptoglobin is α_2_-sialoglycoprotein that shows immunomodulatory properties [7]. By the original method described by Smities [8] Hp displays three common genotypes Hp*1/*1, Hp*2/*1 and Hp*2/*2 due to the presence of two codominant alleles Hp*1 and Hp*2 at an autosomal locus. Haptoglobin is composed of an α chain and a β chain. Hp polymorphism is due to an intragenic duplication in the α chain. Hp *1/*1 polypeptides form a tetramer that migrates as a single band, whereas Hp*2/*1 and Hp*2/*2 polypeptides respectively form various heteropolymers and homopolymers that migrate as a series of slower bands.

Several studies point to clinical relevance of Hp polymorphism in the susceptibility to immunoallergic and infectious diseases. Hp inhibits prostaglandin synthesis [9] and shows antioxidant effects [10] and some properties of Hp suggest that it has a specific role in the modulation of the immune system. Recent studies by Berkova *et al*. [11,12] suggest that Hp may play a role in immunological processes involved in human reproduction.

Olson et al. [2] recently showed that Hp messenger RNA is expressed in implantation-stage rabbit endometrium. Like other anti-inflammatory and immunosuppressive mediators induced by interleukin-6, Hp also appears to negatively regulate the inflammatory response. Therefore, its presence *in utero *at implantation may play an important role in the modulation of the maternal reaction against the blastocyst. Compared with other genotypes, Hp*1/*1 produces more protein and smaller polymers that may diffuse more readily at the site of implantation [13] pointing to a possible explanation for the greater natural fertility of women with Hp*1/*1 genotype compared with women with an Hp*2 allele.

Oxidative damage due to reactive oxygen species is probably responsible for most of the deleterious effects of smoke on susceptibility to severe pathological conditions [14]. Biochemical parameters in smokers show variability among individuals, and this has been attributed to genetic differences. This hypothesis is supported by the fact that several enzymes involved in the protection from oxidative damage show genetic polymorphism [15,16].

It is possible to explain the pattern of interactions presently described among Hp, smoke and fertility on the basis of oxidative damage by smoke and of a relative higher protection from oxidative damage by haptoglobin *1/*1 genotype. However, we propose an alternative explanation. Hp*1/*1 produces smaller polymers which may diffuse more readily at the site of implantation, exerting a favorable effect on reproductive success. Smoking increases the production of haptoglobin [3], and our data on the appearance of Hp in the early stage of extrauterine life suggest that the effect of smoking on the production of Hp may depend on Hp genotype, being more marked (or perhaps specific) for Hp*1/*1.

Several studies have shown an association between Hp phenotype and psychiatric disorders suggesting a possible link with drug addiction (17, 18, 19). Neither in Rome and in Penne, however, we have found a direct significant association between smoking habit and Hp phenotype.

The interactions between Hp genotype and smoke on the rate of Hp production are worth further investigation in view of the possible clinical relevance especially in immunologic and infectious diseases involving the respiratory apparatus.

## Competing interests

The authors declare that they have no competing interests.
